# Assessing the feasibility of interrupting the transmission of soil-transmitted helminths through mass drug administration: The DeWorm3 cluster randomized trial protocol

**DOI:** 10.1371/journal.pntd.0006166

**Published:** 2018-01-18

**Authors:** Kristjana Hrönn Ásbjörnsdóttir, Sitara S. Rao Ajjampur, Roy M. Anderson, Robin Bailey, Iain Gardiner, Katherine E. Halliday, Moudachirou Ibikounle, Khumbo Kalua, Gagandeep Kang, D. Timothy J. Littlewood, Adrian J. F. Luty, Arianna Rubin Means, William Oswald, Rachel L. Pullan, Rajiv Sarkar, Fabian Schär, Adam Szpiro, James E. Truscott, Marleen Werkman, Elodie Yard, Judd L. Walson, The DeWorm3 Trials Team

**Affiliations:** 1 DeWorm3, Division of Life Sciences, Natural History Museum, London, United Kingdom; 2 Department of Global Health, University of Washington, Seattle, United States; 3 Division of Gastrointestinal Sciences, Christian Medical College, Vellore, India; 4 London Centre for Neglected Tropical Disease Research, Department of Infectious Disease Epidemiology, School of Public Health, St. Marys Campus, Imperial College London, London, United Kingdom; 5 Clinical Research Department, London School of Hygiene & Tropical Medicine, London, United Kingdom; 6 Faculty of Infectious and Tropical Diseases, London School of Hygiene and Tropical Medicine, London, United Kingdom; 7 Département de Zoologie, Faculté des Sciences et Techniques, Université d'Abomey-Calavi 01BP526, Cotonou, Benin; 8 Blantyre Institute for Community Outreach, Lions Sight First Eye Hospital, Blantyre, Malawi; 9 MERIT UMR 216, Institut de Recherche pour le Développement, Paris, France; 10 Department of Biostatistics, University of Washington, Seattle, United States; Ministère de la Santé Publique et de la Lutte contre les Endémies, NIGER

## Abstract

Current control strategies for soil-transmitted helminths (STH) emphasize morbidity control through mass drug administration (MDA) targeting preschool- and school-age children, women of childbearing age and adults in certain high-risk occupations such as agricultural laborers or miners. This strategy is effective at reducing morbidity in those treated but, without massive economic development, it is unlikely it will interrupt transmission. MDA will therefore need to continue indefinitely to maintain benefit. Mathematical models suggest that transmission interruption may be achievable through MDA alone, provided that all age groups are targeted with high coverage. The DeWorm3 Project will test the feasibility of interrupting STH transmission using biannual MDA targeting all age groups. Study sites (population ≥80,000) have been identified in Benin, Malawi and India. Each site will be divided into 40 clusters, to be randomized 1:1 to three years of twice-annual community-wide MDA or standard-of-care MDA, typically annual school-based deworming. Community-wide MDA will be delivered door-to-door, while standard-of-care MDA will be delivered according to national guidelines. The primary outcome is transmission interruption of the STH species present at each site, defined as weighted cluster-level prevalence ≤2% by quantitative polymerase chain reaction (qPCR), 24 months after the final round of MDA. Secondary outcomes include the endline prevalence of STH, overall and by species, and the endline prevalence of STH among children under five as an indicator of incident infections. Secondary analyses will identify cluster-level factors associated with transmission interruption. Prevalence will be assessed using qPCR of stool samples collected from a random sample of cluster residents at baseline, six months after the final round of MDA and 24 months post-MDA. A smaller number of individuals in each cluster will be followed with annual sampling to monitor trends in prevalence and reinfection throughout the trial.

**Trial registration:**

ClinicalTrials.gov NCT03014167

## Author summary

Soil-transmitted helminths (STH) affect 1.45 billion people worldwide, and high intensity infections are associated with anemia, undernutrition and impaired cognition, particularly among children. Mathematical models suggest it may be possible to interrupt the transmission of STH in a community by expanding mass drug administration (MDA) from targeted high-risk groups (primarily school-aged children and women of child-bearing age) to all community members with high coverage. The DeWorm3 Project will test the feasibility of this approach to interrupting the transmission of STH using a series of cluster randomized trials in Benin, India and Malawi. Each study area (population ≥80,000) will be divided into 40 clusters and randomized to community-wide or standard-of-care targeted MDA for three years. Two years following the final round of MDA, prevalence of STH will be compared between arms and transmission interruption assessed in each cluster. The DeWorm3 trials will provide stakeholders with information regarding the potential to switch from STH control to a more ambitious and sustainable strategy.

## Introduction

The soil-transmitted helminths (STH) are group of intestinal parasites (*Ascaris lumbricoides*, *Ancylostoma duodenale*, *Necator americanus* and *Trichuris trichiura)* targeted for control by the London Declaration. These parasites infect an estimated 1.45 billion people globally, resulting in the loss of almost 5 million disability adjusted life years (DALYs) annually [1, 2]. High to moderate intensity STH infections are associated with increased risk of malnutrition, iron-deficiency anemia and other adverse physical and cognitive morbidities, particularly in children. The current World Health Organization (WHO) strategy for controlling STH is based on mass drug administration (MDA) of albendazole or mebendazole to pre-school and school-age children (PSAC and SAC), women of childbearing age (including pregnant women in the second and third trimesters and breastfeeding women) and adults in certain high-risk occupations such as agricultural laborers or miners, with the goal of eliminating STH-related morbidity [3–5].

While the current strategy of MDA for STH control has proven successful in controlling morbidity, the incidence of reinfection post-treatment is high and targeted MDA is unlikely to break transmission of STH in many settings[6]. This is in large part due to adult reservoirs of disease [7, 8]. Even if coverage of all targeted populations were optimized, the current STH strategy would likely need to be continued until significant economic development and universal water, sanitation and hygiene (WASH) access are able to interrupt transmission[9]. As a result, there is increased interest in alternative strategies to interrupt STH transmission using broadly administered MDA strategies targeting all age groups [10], which a recent systematic review and meta-analysis showed are more effective in reducing the prevalence of STH infection[11]. Several trials investigating the differential impact of community-wide versus school-based MDA on STH transmission are currently underway.[12, 13]

Other NTD programs have been successful in achieving high treatment coverage of MDA to entire communities, including adults. For example, programs targeting lymphatic filariasis (LF) elimination have achieved considerable community acceptability and high MDA coverage in many endemic countries. Of the 73 countries endemic for LF in 2014, 18 have discontinued MDA and are in a state of post-MDA surveillance, pending confirmation that LF transmission has been interrupted [14]. Given that LF programs provide five to seven rounds of MDA to entire communities with albendazole (in combination with ivermectin or diethylcarbamazine), there is a unique opportunity to leverage the LF program platform to continue community-wide MDA after programs have proceeded to surveillance and attempt to break the transmission of STH.

The DeWorm3 Project (ClinicalTrials.gov Identifier NCT03014167) will conduct a series of community cluster randomized trials in India, Malawi and Benin in order to determine whether continuing community-wide MDA with albendazole following cessation of LF programs can interrupt the transmission of STH (*Ascaris lumbricoides*, *Ancylostoma duodenale*, *Necator americanus* and *Trichuris trichiura)* in focal geographic areas. Specifically, this project will demonstrate the feasibility of interrupting STH transmission using a strategy of twice-annual community-wide MDA, compared to the current national STH strategy at each site.

### Aims and objectives

The general objective of DeWorm3 is to determine the feasibility of interrupting the transmission of STH (*A*. *lumbricoides*, *A*. *duodenale*, *N*. *americanus* and *T*. *trichiura)* in focal geographic areas in Africa and Asia by expanding the population targeted and the frequency of delivery of MDA with albendazole.

To address this objective, in trial sites in India, Malawi and Benin, where LF programs have delivered at least five rounds of MDA of albendazole (plus ivermectin or DEC), clusters will be randomized to receive twice-annual community-wide MDA or standard of care (current national STH MDA strategy, Table 1) for three years.

**Table 1:** DeWorm3 study sites and national mass drug administration (MDA) strategies for control of soil-transmitted helminths (STH) in each country. Each country’s national guidelines will be followed in the standard of care / control arm of the trial.

Study site	National Standard of Care STH Control Strategy	STH endemicity^1^	Study area
**Benin**	School-based MDA Targets SAC (5–14 years old)	Modeled 2010 national prevalence[2]: Hookworm 1–10% *Ascaris* 1–10% *Trichuris* 1–10%	Commune of Comé, Department of Mono. Consists of Comé town and surrounding rural area.
**India**	School-based MDA and National Deworming Days Targets PSAC and SAC (1–19 years old)	Observed 2013–2014 prevalence in Jawadhu Hills [28]: Hookworm 18.5% *Ascaris* 0.4% *Trichuris* <1%	Thimiri and Jawadhu Hills, State of Tamil Nadu. Largely rural; consists of tribal area in Jawadhu Hills and additional plains area in Thimiri.
**Malawi**	School-based MDA and Child Health Days Targets PSAC and SAC (1–14 years old)	Modeled 2010 national prevalence[2]: Hookworm 1–10% *Ascaris* 1–10% *Trichuris* 1–10%	Mangochi District, Southern Region. Largely rural; a subset of the district (full population 1 million) will be included.

^1^Published estimates available at the time of protocol development; up-to-date baseline prevalences at each site will be measured prior to randomization.

Primary objectives:

1. To compare the prevalence of each STH species *(A*. *lumbricoides*, *A*. *duodenale*, *N*. *americanus* and *T*. *trichiura*) measured by quantitative PCR 24 months after stopping MDA, between clusters randomised to receive twice-yearly community-wide MDA versus clusters randomised to receive standard of care pre-SAC and SAC targeted MDA.
2. To determine whether the transmission of each STH species *(A*. *lumbricoides*, *A*. *duodenale*, *N*. *americanus* and *T*. *trichiura)* can be interrupted using MDA with albendazole, defined as reaching a weighted point prevalence of ≤2% of that species measured by quantitative PCR 24 months after stopping MDA in clusters receiving either twice-yearly community-wide MDA or standard of care pre-SAC and SAC targeted MDA.

Secondary objectives include:

3 To compare the prevalence of each STH species by quantitative PCR in children under 5 years of age by randomization arm 24 months following the last round of MDA.

### Trial summary

**Fig 1:**
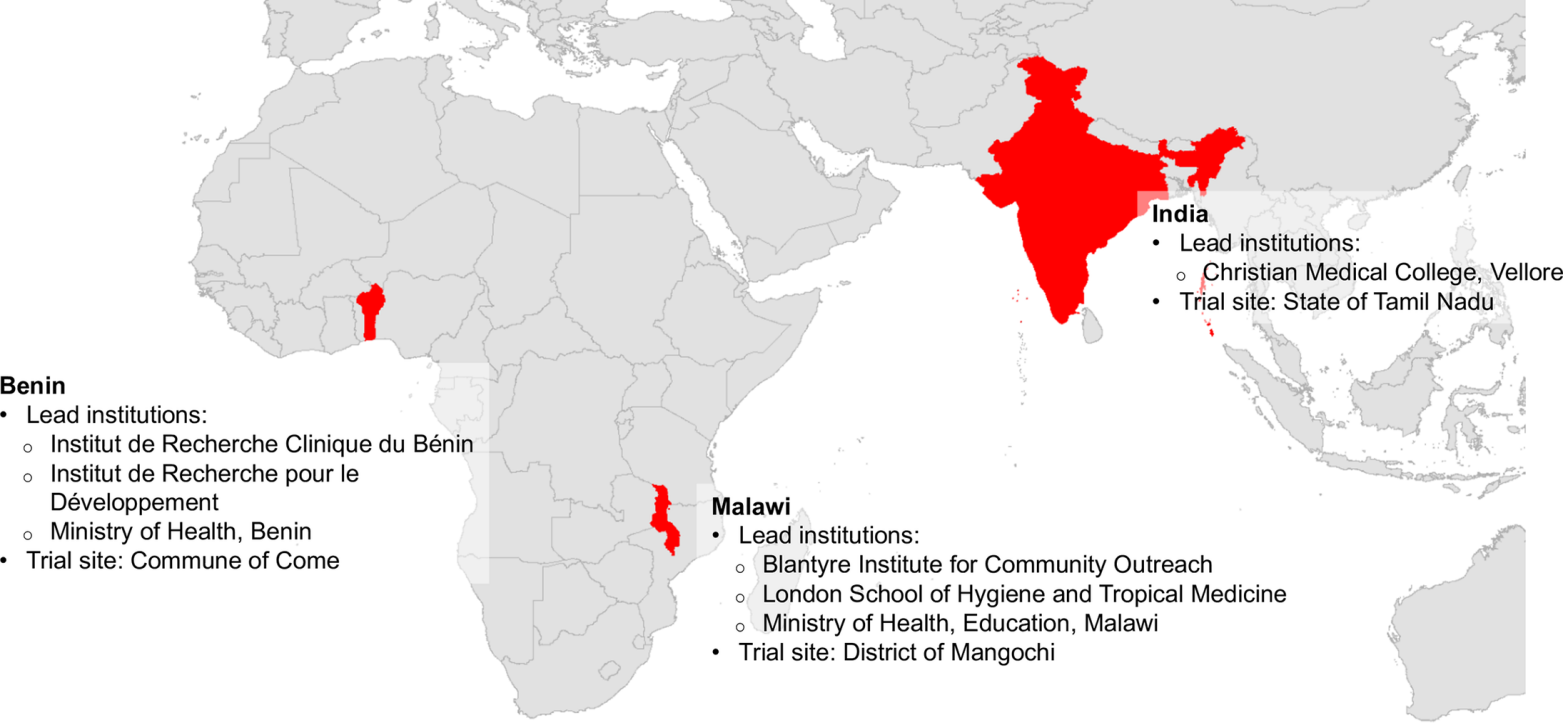
DeWorm3 trial sites and partners. Figure created for DeWorm3 using shapefiles from GADM database of Global Administrative Areas 2012, version 2.0 (gadm.org) and ArcGIS Desktop: Release 10 (Redlands, CA).

The DeWorm3 field trials are a series of community cluster randomized trials conducted in focal geographic areas in Benin, India and Malawi (Fig 1) comparing twice-annual community-wide MDA to the current standard of care MDA (typically school-based deworming) for interruption of STH transmission. The study is summarized in Table 2. Each study site includes a minimum total population of 80,000 individuals, to be divided into 40 total clusters with a minimum population of 1,650 individuals in each. Clusters may consist of one or more villages, settlements or zones within urban areas, and cluster boundaries will take into account administrative and / or geographic barriers. Clusters will be randomized 1:1 to three consecutive years of either;

1. Twice-annual community-wide MDA (intervention arm), or
2. Targeted MDA (control, standard-of-care arm) delivered in accordance with WHO recommendations and national Ministry of Health (MOH) guidelines.

**Table 2:** Summary of the DeWorm3 field trials.

**Study Design:**	Community cluster randomized controlled trial
**Intervention:**	Twice-annual community-wide MDA of albendazole for 3 years delivered to all individuals over 24 months of age
**Control:**	Targeted MDA of PSAC and SAC with albendazole for 3 years delivered in accordance with national MOH guidelines
**Primary Outcome:**	Transmission interruption of STH species (weighted prevalence ≤2% 24 months following the final round of MDA)
**MDA eligibility criteria:**	A PSAC or SAC residing in a control cluster OR any resident in an intervention cluster Eligible for treatment with albendazole according to WHO and national guidelines
**MDA exclusion criteria:**	Children under one or two years of age, depending on national guidelines Women in the first trimester of pregnancy History of adverse reaction to benzimidazoles Acutely ill at the time of MDA
**Sampling eligibility criteria:**	Residents of the study cluster based on census and questionnaire responses Provide consent and/or assent (as applicable)
**Sampling exclusion criteria:**	Individuals who do not reside in the study cluster Refusal to consent/assent Enrollees in the longitudinal monitoring cohort ineligible for inclusion in cross-sectional sampling
**Sampling schedule:**	Cross-sectional sampling of randomly selected eligible participants will take place at baseline (pre-MDA), post-treatment (six months following the final round of MDA), and at endline (24 months following the final round of MDA) Longitudinal monitoring cohorts will be sampled annually

Randomization of clusters to community-wide vs. targeted MDA will be stratified by site, and restricted randomization will be performed to ensure balance in baseline factors between arms; these may include population, age distribution, socio-economic status (SES), WASH access and urban / rural designation of clusters. Baseline prevalence may be included in the restricted randomization, depending on the degree of variability between clusters. A list of restricted randomization scenarios will be generated by the DeWorm3 Clinical Trial and Implementation Science Support Unit, with the final scenario randomly selected by each site team.

#### Primary outcome

Transmission interruption is defined as a cluster-level point prevalence ≤2% of the dominant STH species (primary outcome) and any detectable STH species (secondary outcome) at the study endpoint, measured by qPCR 24 months after the final round of MDA. The prevalence threshold and post-treatment interval were selected based on STH transmission models to maximize positive predictive value (PPV), defined here as the proportion of communities below the threshold in which STH prevalence will progress to elimination rather than revert to pre-MDA levels after drug pressure is removed. In mathematical models, the PPV of different transmission thresholds varies by STH species and pre-MDA transmission intensity / starting prevalence, the size of the community, and the post-treatment follow-up interval. An absolute prevalence threshold of 2% at 24 months post-MDA has a PPV of >0.8 for most scenarios explored in the models, meaning that 80% of communities that attain the threshold for each individual STH species are expected to progress to elimination.[15, 16]

#### Mass drug administration

In the intervention arm, all individuals 24 months of age and older will be eligible for treatment with albendazole in accordance with WHO guidelines, with the exception of women in their first trimester of pregnancy, anyone with a history of adverse reactions to benzimidazoles, and those acutely ill at the time of MDA (Table 2). All eligible individuals will receive a single dose of albendazole during twice-annual campaigns. House-to-house MDA will be conducted and study staff or village volunteers will directly observe MDA compliance; where directly observed treatment is not possible, this will be recorded. Mop-up campaigns targeting untreated individuals will be conducted in all clusters.

In control clusters, all those targeted by existing national guidelines (primarily PSAC and SAC) will be eligible for treatment. MDA will be provided through the existing National STH Program or a similar distribution structure. Eligible individuals will receive a single dose of albendazole through school-based distribution or during alternative MDA campaigns, according to site-specific national guidelines. Non-enrolled SAC and PSAC will be encouraged to come to school for treatment on deworming days, or will be reached by strategies appropriate to the local context in collaboration with local ministries and authorities. MDA will be administered by teachers, health workers or volunteers, according to national practice.

In order to encourage high treatment coverage in the community-wide intervention arm, enhanced community sensitization activities will take place within one month of scheduled MDA events. Sensitization activities will be designed in close consultation and collaboration with the National NTD program and in accordance with site norms and past MDA experiences. Where possible, MDA activities will be scheduled to accommodate seasonal and cultural activities that might influence MDA participation, such as holidays, migratory events, or crop harvesting. In the control arm, standard sensitization activities will take place according to national guidelines.

### Data collection

**Fig 2:**
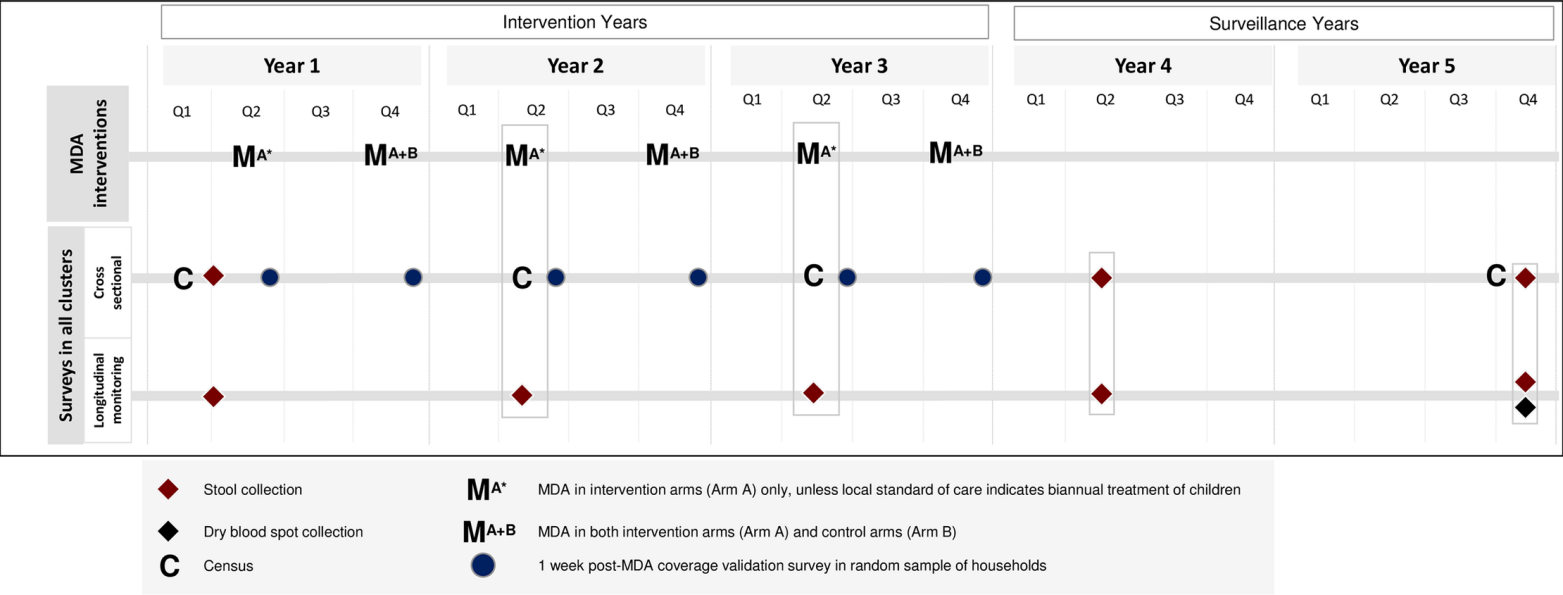
Schematic of planned data and sample collection time points for the DeWorm3 field trials.

All sites will collect information on a core set of variables with standard definitions. Data collection instruments were developed with input from all sites and will be translated into local languages. Data will be collected electronically, using Android devices equipped with SurveyCTO software (Dobility, Inc; Cambridge, MA, USA and Ahmedabad, India). Data collected at each site will be maintained in a harmonized central database with site-specific and central quality control procedures. Data collection activities are summarized in Fig 2.

#### Data access

Data collected at each site will be owned by the site’s Project Leads and National Government and maintained on a site-specific, password-protected SurveyCTO server. Pooled, de-identified data from all sites will be maintained on a central, password-protected SurveyCTO server by the DeWorm3 Project. Access to the Project data will be granted by the Project Leads at each site and the Project PI.

#### Study area census

An initial baseline census of all individuals residing within the boundaries of each study area will be performed, enumerating and collecting basic sociodemographic information on the inhabitants of each household along with the GPS coordinates of their dwellings. Up to three attempts will be made to reach each household for inclusion. The baseline census will provide information on population and STH risk factors to inform initial cluster randomisation. Census updates will then be conducted annually to verify births, deaths, and migration status of household members. Censuses will enable random sampling of participants for outcome assessment, provide denominator data for MDA coverage assessments, and be used to quantify in- and out-migration. Migration-specific questions on the census will also be used to determine when mop-up MDA activities will occur in a given cluster.

#### Prevalence assessments

**Fig 3:**
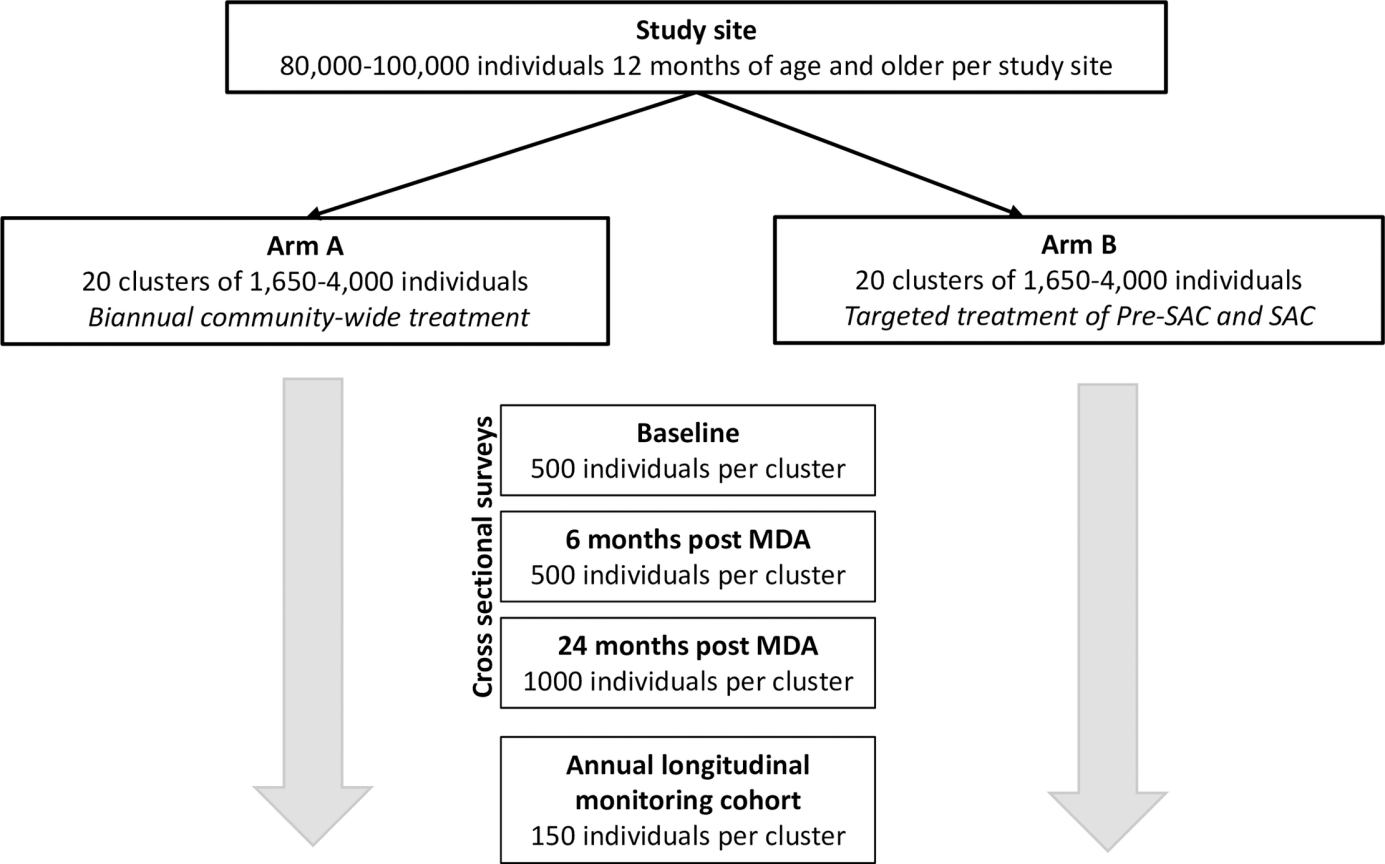
Flow chart showing outcome assessment in the DeWorm3 field trials.

Cross-sectional assessments of STH prevalence and intensity will take place via random sampling from the most recent census. Cross-sectional surveys take place at baseline (pre-MDA), post-intervention (6 months following the final round of MDA), and endline (24 months following the final round of MDA). Five hundred people will be sampled per cluster at baseline and post-intervention, while at endline, 1000 people per cluster will be sampled (Fig 3). Up to three attempts will be made to reach each individual for the purposes of the cross-sectional prevalence assessments. Cross-sectional assessment data will be used to inform the study’s primary aims, measuring endline STH prevalence and determining whether or not transmission interruption has been achieved at a cluster-level. Stool samples will be collected for DNA extraction and qPCR analysis; cycle threshold will be used to assess sample positivity and as a proxy for intensity of infection. Microscopic examination using Kato-Katz technique will be performed to assess presence and intensity of infection on a subset of 100 samples per cluster at the post-intervention and endline surveys, while baseline Kato-Katz assessment will come from the Longitudinal monitoring cohorts (see below).

#### Longitudinal monitoring cohorts

At baseline, a subset of 150 individuals in each cluster will be invited to participate in longitudinal monitoring cohorts for the duration of the trial. The longitudinal cohort will be selected at random from an age-stratified census (30 PSAC, 30 SAC, 90 adults) and participants will be excluded if they report that they plan to move out of the study area within the study period. These individuals will provide stool samples annually for immediate analysis using Kato-Katz and whole stool samples will be stored for subsequent DNA extraction and testing via qPCR. Data collected at interim timepoints in these cohorts will serve to assess rates of change in STH prevalence, quantify reinfection after a negative test where applicable, and to validate and refine mathematical models. Individuals who are part of the longitudinal monitoring cohorts will not be eligible to provide samples for cross-sectional sampling.

#### Coverage surveys

A random sample of 50 households in each cluster will be invited to participate in a post-MDA coverage survey within one week of community-wide or standard of care MDA taking place in that cluster. Coverage surveys will be conducted prior to any cluster-wide mop-up activities in the intervention arm. All individuals in the selected household will be invited to participate in the coverage survey, regardless of whether they were intended recipients of MDA. A subsample of those participants surveyed within 72 hours of MDA in their clusters will be asked to provide a urine sample for coverage validation by detection of albendazole metabolites. Treatment coverage is defined as the proportion of eligible individuals who were reached by MDA activities and who ingested albendazole during MDA; coverage of the intervention and adherence to treatment will be assessed separately. Questions in the survey align with WHO-endorsed coverage survey for use by national programs[17]. In accordance with NTD coverage survey guidelines, the individuals who conduct the coverage survey in a given cluster will not be the same individuals who delivered the drugs [18].

#### School facility surveys

An annual school facility survey will also be conducted to assess the presence of water and sanitation facilities at schools in all clusters and to determine the enrollment and attendance rates of children in the study site. These data provide an opportunity to validate the annual census and to understand the totality of children’s risk for STH acquisition.

### Study management and coordination

Principal Investigators (PIs) at each site are responsible for the conduct of trial procedures, with central coordination of trial activities by the Project PI and team at the Natural History Museum in London. The DeWorm3 coordinating team at the Natural History Museum is supported by five units: (1) the Clinical Trial and Implementation Science Support Unit at the University of Washington, USA, (2) the Modeling and Trial Simulation Support Unit at Imperial College London, (3) the Economic Analysis Support Unit at Swiss Tropical and Public Health Institute, (4) the Trial Conduct and Coordination Support Unit, London Applied & Spatial Epidemiology Research Group (LASER), London School of Hygiene & Tropical Medicine; and (5) the STH Molecular Diagnostics Support Unit at Smith College, USA and the Christian Medical College, Vellore, India.

A community advisory board (CAB) will be established at each study site and designed in accordance with site preferences in order to guide and inform appropriate implementation of all study procedures and engagement with community members.

A four-member Data Safety and Monitoring Committee (DSMC) has been formed to evaluate the statistical analysis plan and monitor study progress and severe adverse events (SAEs). MDA programs distributing albendazole will conduct passive adverse event (AE) monitoring and reporting. SAEs, including all deaths and any events classified as “definitely”, “probably” or “possibly” related to the study drug according to WHO criteria, will be reported to the DSMC and to all appropriate regulatory bodies within 48 hours of the study team becoming aware of them.

### Ethics statement

The DeWorm3 Project has been reviewed and approved by the Institut de Recherche Clinique au Bénin (IRCB) through the National Ethics Committee for Health Research (002-2017/CNERS-MS) from the Ministry of Health in Benin, The London School of Hygiene and Tropical Medicine (12013), The College of Medicine Research Ethics Committee (P.04/17/2161) in Malawi and the Christian Medical College Institutional Review Board in Vellore, India (10392). The study was also approved by The Human Subjects Division at the University of Washington (STUDY00000180).

#### Consent procedures

Data collectors will obtain consent / assent as appropriate prior to all data collection activities. Written informed consent will be sought if the individual can write, while for any individual who cannot write, oral consent will be given in the presence of a witness and documented with a thumbprint. The use of oral consent in these circumstances has been approved by the relevant ethical committees.

The head of household or other adult member of the household will give informed consent for participation in the baseline census and annual updates. Participants in each round of cross-sectional sampling and in the coverage survey will give informed consent for their participation. Participants in the longitudinal monitoring cohorts will provide consent for repeated sample collection and repeated rounds of data collection at enrollment. If a child is selected for any of the above activities, parents or guardians will give informed consent on behalf of the child; children will provide assent in accordance with national ethical guidelines. Consent for participation in the school facility survey will be sought from head teachers or others with decision-making authority on behalf of each school. Approval to approach each school will be sought from Ministries of Education if required by each country’s guidelines.

#### Treatment of STH infections identified by Kato-Katz

Following cross-sectional and longitudinal surveys, all individuals in the intervention arm with STH infections identified using Kato-Katz will be treated during community-wide MDA. All PSAC and SAC in the control arm with identified infections will be treated during targeted MDA. WHO guidelines do not recommend MDA for adults; however, any adult (≥15 years of age) in control clusters found by Kato-Katz a) to have moderate to heavy intensity STH infection according to WHO definitions[19] or b) to require treatment according to each country’s guidelines will be provided treatment by study staff.

### Sample collection and analysis procedures

#### Sample collection and storage

Consenting participants will be provided plastic containers for collection of a stool sample, to be submitted within 24 hours. Stool will be collected from households or, if necessary, delivered to a central location, depending on participant preference. Stool samples will be divided into aliquots for microscopic examination, DNA extraction, and storage as appropriate. Stool samples intended for DNA extraction and stored for subsequent analyses will be cryopreserved at -80°C.

Participants in the longitudinal cohort will have finger prick blood samples taken at the final round of follow-up to contribute whole blood, stored as dried blood spots on filter paper for evaluation of antigen detection of infection and measurement of immune response to STH infection and clearance. Future applications to relevant ethical review committees will be made for any unspecified analyses.

Participants in the post-MDA coverage surveys who are selected for the coverage validation sub-study will be provided with plastic containers and asked to provide a urine sample at the time of the survey. Samples will be cryopreserved at -20°C for subsequent analyses.

#### Laboratory procedures

Aliquots of all stool samples will be stored for parasite DNA extraction and qPCR analysis to detect the presence and intensity of infection with each individual species of STH [20]. All sites will ship duplicates of 10% of all study samples to the central laboratory facility at Christian Medical College (CMC) in Vellore, India for quality assurance and control. CMC will in turn ship 10% of samples to Smith College, Massachusetts, USA for quality assurance and control. A subset of samples positive by qPCR will be stored for subsequent screening for anthelminthic resistance mutations.

A second aliquot of samples selected for Kato-Katz will be analyzed in duplicate by microscopy. These aliquots will be analyzed within 24 hours of collection and within 30 minutes of sample preparation, to prevent the degradation of hookworm eggs. Two slides will be prepared and analyzed per specimen. Ten percent of slides will be submitted for quality control checks to be performed by laboratory supervisors. Laboratory personnel will be blinded to treatment allocation during assessment.

Urine samples collected for validation during the post-MDA coverage survey will be analyzed using high performance liquid chromatography (HPLC) for the detection of albendazole metabolites.

### Statistical analysis plan

Primary analyses will be intent-to-treat and conducted separately for each site, unless otherwise specified. Missing data on individual-level characteristics will be estimated using multiple imputation.

#### Primary objectives

The effect of the primary exposure, MDA strategy, on endline infection with each STH species (Objective 1) will be analyzed using generalized estimating equations with binomial family and exchangeable correlation matrix. The primary exposure variable will be randomization arm, while the primary outcome will be individual-level STH infection status (positive or negative for each STH species by qPCR). Models may be adjusted for baseline cluster-level STH prevalence and SES, WASH access, age, gender of the participant, household size, or other relevant variables as appropriate; both adjusted and unadjusted estimates will be presented. In the primary analysis, data will be analyzed separately by site, while a secondary analysis will pool all data and test for effect modification by study site using an interaction term.

Transmission interruption in a cluster (Objective 2) will be defined as achieving a prevalence of each STH species of ≤2% with 95% confidence using a one-sided binomial test, by qPCR 24 months after the final round of MDA. Measured endline prevalence will be weighted by age and sex to reflect the underlying cluster population. The effect of the primary exposure on successful transmission interruption will be calculated by comparing the proportion of clusters in each arm in which transmission is successfully interrupted using a chi-squared test.

#### Secondary objectives

The effect of MDA strategy on endline infection with any STH species overall among children born during the trial (Objective 3) will be analyzed using generalized estimating equations with binomial family and exchangeable correlation matrix, as detailed above. Models may be adjusted for baseline cluster-level STH prevalence and SES, WASH access, age, gender of the participant, household size, or other relevant variables as appropriate.

#### Secondary analyses

The effect of MDA strategy on treatment coverage a) overall and b) among SAC and PSAC will be analyzed using self-reported coverage (defined as receiving and ingesting albendazole during the timeframe of the most recent round of MDA) from post-MDA coverage surveys in each arm. The effect of randomization arm on self-reported coverage will be analyzed using generalized estimating equations with binomial family and exchangeable correlation matrix and may be adjusted for age, gender, current school attendance, and study year or other relevant variables as appropriate.

Cluster-level correlates of STH transmission interruption (as defined by reaching a cluster prevalence of each STH ≤2% 24 months after stopping MDA) will be assessed using logistic regression. Key correlates of breaking the transmission of STH will include baseline prevalence and species-specific distribution of STH infection, age distribution, population migration levels, treatment coverage and compliance, rates of open defecation and population density.

### Sample size estimates

Study areas in each country will have a minimum population of 80,000 people, and clusters will have a minimum population of 1,650 individuals. The minimum cluster size was selected to reduce the probability of repeatedly sampling the same individuals at all three cross-sectional time points to <10%, allowing for 10% refusal rate and the exclusion of the longitudinal monitoring cohort from the eligible population.

#### Power for primary objectives

For Primary Objective 1, comparing the endline prevalence of each STH species at each site, power calculations for a range of scenarios were conducted. The following formula[21]:

$$c=1+(Z_{\propto /2}+Z_{\beta }{)}^{2}\frac{{[\pi }_{0}(1-\pi_{0})+\pi_{1}(1-\pi_{1})][1+(m-1)\rho ]}{m{(\pi }_{0}-\pi_{1}{)}^{2}}$$

was used to calculate the prevalence in the control clusters (*π*_0_) that would enable detection of a difference between arms with 80% power, given an endline prevalence in the intervention clusters (*π*_1_) of 2% and a range of assumptions of intracluster correlation coefficient (*ρ* = 0.003 to *ρ* = 0.005), number of clusters per arm (*c* = 15 to *c* = 20) and number of people sampled per cluster (*m* = 500 or *m* = 1,000), assuming α = 0.05. The detectable alternative *π*_0_ ranged from 3% to 3.5% (data not shown).

Power to detect differences in final prevalence between arms given plausible values for *π*_0_, ranging from 7% to 10%, was then estimated using simulations conducted in R (R Center for Statistical Computing, Vienna. Austria). Each simulation assumed 1,000 individuals per cluster and a binomial distribution of STH prevalence with a mean of 2% in the intervention clusters and *π*_0_ in the control arm, a range of ICC and α = 0.05; 10,000 repetitions were run for each scenario. Power was ≥98% for all scenarios simulated.

Power for Primary Objective 2, to detect transmission interruption and differences in the proportion of clusters where transmission is interrupted by arm, was also estimated using simulation in R. The threshold for transmission interruption was set at 2% prevalence, and a cluster was considered to have interrupted transmission if prevalence could be declared to be below the threshold with 95% confidence, using a one-sided binomial test. Simulations estimated power to detect a difference in the proportion of clusters in which transmission was interrupted by arm.

**Fig 4:**
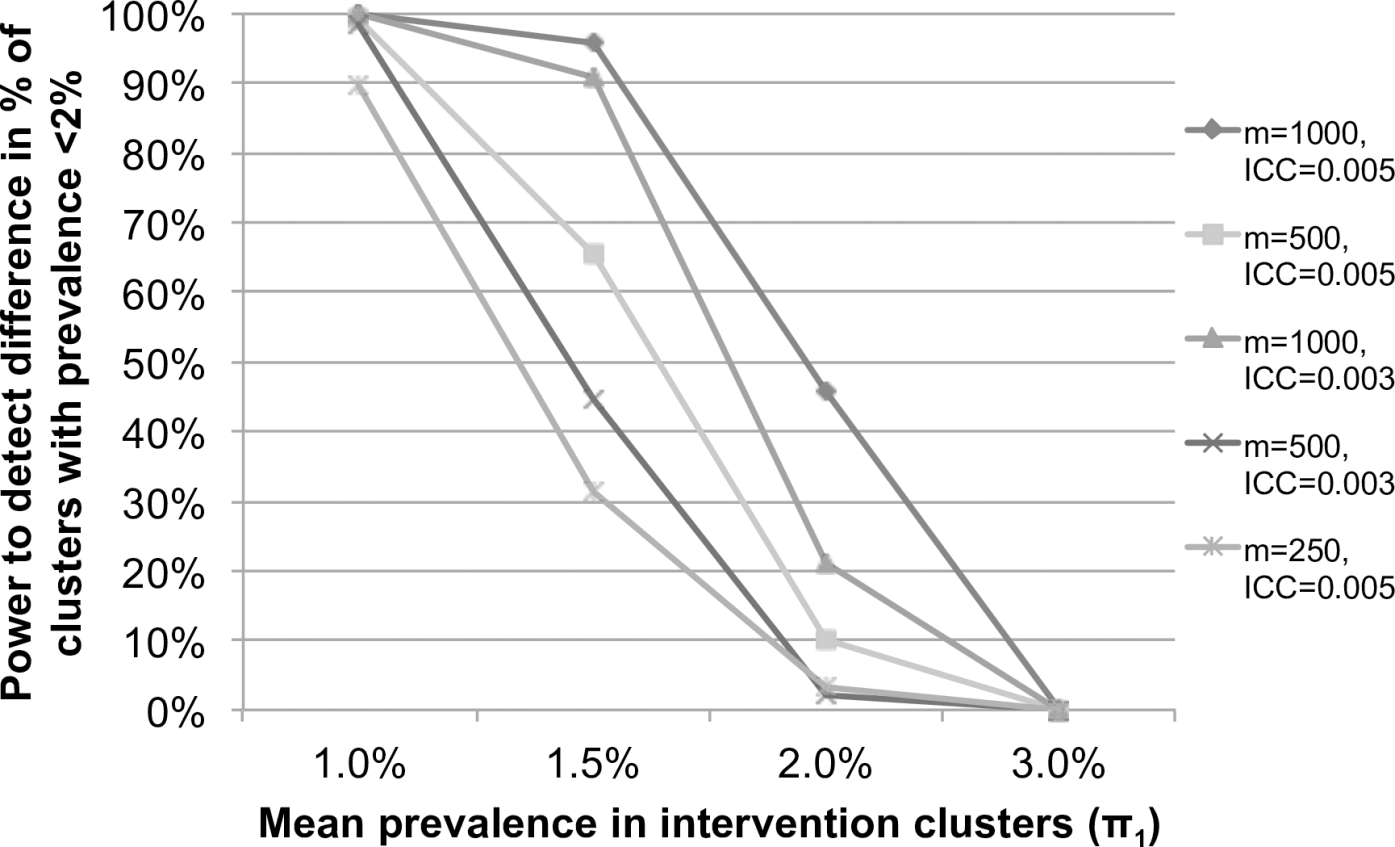
Power to detect a difference in the proportion of clusters achieving transmission interruption (Primary Objective 2) assuming 7% mean prevalence in the targeted clusters (π_0_), by mean endline prevalence in the intervention clusters (π_1_), intracluster correlation (ICC) and number of people measured per cluster (m), estimated by simulation.

Simulations assumed 20 clusters per arm, 500 or 1,000 individuals per cluster (*m*), and a binomial distribution of STH prevalence with a mean of 7% in the targeted arm (*π*_0_) and *π*_1_ in the intervention arm, a range of ICC (*ρ* = 0.003 or *ρ* = 0.005), and α = 0.05; 10,000 repetitions were run for each scenario. For a fixed mean endline prevalence of 7% in the targeted arm (*π*_0_), power to detect a difference in the proportion of clusters in which transmission is interrupted by arm varies by number of individuals sampled per cluster, ICC and endline prevalence in the intervention arm (*π*_1_), as shown in Fig 4.

These power calculations demonstrate that with 20 clusters per arm, evaluating the STH infection status of 500–1,000 people per cluster at endline would provide >80% power for the primary objectives in most scenarios. Thus, a target sample size of 500 individuals at baseline and post-intervention (6 months post-MDA) and 1,000 individuals at endline (24 months post-MDA) was chosen per cluster for the cross-sectional prevalence assessments.

### Potential risks and challenges

A major risk to the DeWorm3 Project is low treatment coverage and/or adherence, particularly if the same individuals are repeatedly missed [15, 22, 23]. Low coverage could be a result of failure to access sufficient proportions of the population or suboptimal adherence (i.e. participants do not consume the drugs they are given), or be due to high rates of population migration. Migration could further pose risks to the project due to potential importation of infection from outside of the study area. Although not applicable to the sites selected for DeWorm3, a high baseline prevalence of *Trichuris* infection was recognized as a significant risk to an MDA-only strategy for transmission interruption during the planning stages of the study. Owing to the low cure rate of *Trichuris* with albendazole, transmission interruption through MDA in such a setting would be particularly challenging and would likely require use of combination therapy[24, 25]. Reduced cure rates due to resistance mutations are also a concern; STH resistance to benzimidazole anthelminthics is commonly detected in animal samples and resistance mutations have been documented in parasites infecting humans [26, 27]. The emergence of resistance in the context of expanding MDA to the full population could pose a risk to the success of transmission interruption, and will be monitored in samples collected during the study. Other potential challenges include political instability, the presence of transmission hotspots in areas with low coverage, and unprogrammed MDA activities in either trial arm.

## Conclusion

If the DeWorm3 field trials demonstrate that transmission is successfully interrupted at one or more sites, this will provide evidence of the feasibility of an MDA-based approach to STH elimination. Secondary analyses will identify cluster-level factors associated with successful interruption and will provide evidence of the effect of community-wide MDA on prevalence and infection intensity, reinfection rates and the emergence of benzimidazole resistance. The DeWorm3 field trials will provide stakeholders with information to evaluate the potential to switch from a control to an elimination strategy and to optimize future programs and policies to achieve maximum impact.

**Editor's Note:** Please note that PLOS Neglected Tropical Diseases does not customarily publish standalone protocols; however, we are pleased to present this protocol as a complement to the research articles in the DeWorm3 collection.
