# Correction: Assessing the feasibility of interrupting the transmission of soil-transmitted helminths through mass drug administration: The DeWorm3 cluster randomized trial protocol

**DOI:** 10.1371/journal.pntd.0006253

**Published:** 2018-01-31

**Authors:** The PLOS Neglected Tropical Diseases Staff

The affiliation for the eleventh author is incorrect. The affiliation for Adrian J. F. Luty should appear as: MERIT, IRD, Université Paris 5, Sorbonne Paris Cité, Paris, 75006, France. The publisher apologizes for this error.
